# Sustainable Healthcare to Provide Quality Care in the Surgical Industry in the United Kingdom

**DOI:** 10.7759/cureus.38156

**Published:** 2023-04-26

**Authors:** Fathima S Mubarak

**Affiliations:** 1 Cardiothoracic Surgery, Harefield Hospital, Harefield, GBR

**Keywords:** healthcare system, operating theater, financial sustainability, quality assessment in healthcare, uk - united kingdom, environmental sustainability, hospital sustainability, surgical specialty

## Abstract

The surgical industry makes a major contribution to sustainable healthcare. This article aims to critically evaluate sustainable healthcare to provide quality surgical care in the United Kingdom.

For this study, a systematic review was conducted using peer-reviewed studies and articles from the United Kingdom related to surgical and anesthetic fields that were published within the last five years. The journal articles were selected based on their relevance to the sustainability and performance of the healthcare system, including risks, and subsequently screened using the Preferred Reporting Items for Systematic Reviews and Meta-Analyses 2020 model screening approach. The findings of the relevant journal articles were then critically evaluated for each theme.

A total of 79 studies were retrieved, and 15 of the retrieved studies met the inclusion criteria. Of those, 10 articles evaluated existing sustainability practices, only seven articles discussed significant determinants of quality healthcare, and only 86.67% of the articles highlighted the implications of sustainability. The key predictors of high-quality medical care are effective resource management, the acquisition of a moral surgical team, the provision of professional services, integration, short hospital stays, and low mortality and morbidity rates. Conserving water, optimizing treatment routes and transportation, and creating cultural change were found to be the pillars of high-quality, sustainable healthcare.

The concept of sustainability varied between these studies, and limitations on sustainability as a result of reduced mortality, morbidity, and business services were observed. Anesthetic gas emissions from operating rooms continue to have the most detrimental effect on the sustainability of the surgical industry. A significant gap was noted between the available data and their implications.

## Introduction and background

The global economy devotes 10% of its resources to healthcare, and this percentage is constantly rising. The majority of the expensive, technologically advanced medications and interventions are more harmful to humans and the environment than they are beneficial to patients [1]. According to the Academy of Medical Royal Colleges, “sustainable healthcare involves ensuring the ability to provide quality care for future generations by balancing the economic, environmental, and social constraints and demands within healthcare settings. A sustainable healthcare system maintains population health, reduces disease burden, and minimizes healthcare service use” [2].

Healthcare practices have an impact on the environment in several ways, including greenhouse gas emissions, air pollution, ozone depletion, resource shortages, antibiotic footprints, etc. The majority of the aforementioned issues are caused by the surgical department, which is the hospital’s most resource-intensive department and produces 14% of the hospital’s medical waste. Currently, very little is known about the attitudes and actions of surgeons in relation to climate change or the perceived barriers to sustainable practice [3].

Aim and objective

It was discovered that the available data and the implications of the investigations did not align. The crucial determinants of high-quality medical care have been evaluated in a limited number of studies. Therefore, the purpose of this study is to address the need for adequate information on how sustainable healthcare will result in higher-quality surgical care in the United Kingdom. The goal is to critically examine the surgical industry in the United Kingdom in terms of quality care and sustainable healthcare.

This article was previously presented orally at the Association of Surgeons in Training (ASiT) 47th Annual Conference in Liverpool, England, on March 4, 2023.

## Review

Methodology

Research Strategy

The secondary data approach was used, and the research was based on readily available and accessible journal articles with the same scope and objectives and filed under the same research topic. This systematic review was written in accordance with the Preferred Reporting in Systematic Reviews and Meta-Analyses (PRISMA) statement.

Scope

**Figure 1:**
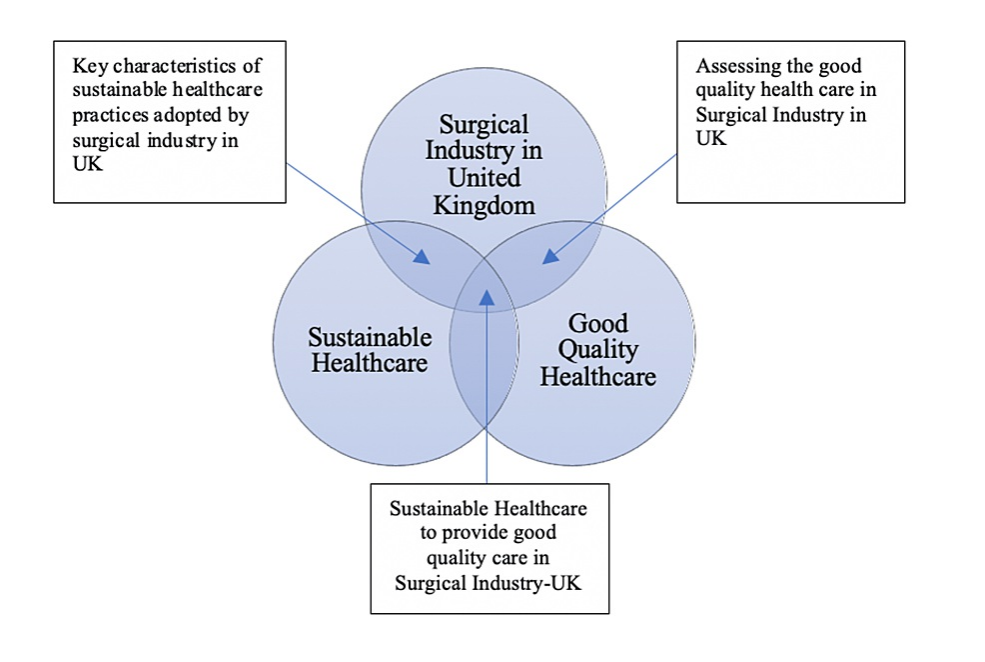
Venn diagram.

This study focused on healthcare sustainability, high-quality healthcare, and the surgical industry in the United Kingdom. These three intersections were used to create a Venn diagram to aid in gaining a better understanding of the scope and in selecting relevant journal articles (Figure 1).

Search Strategy

This stage corresponds to the intersections depicted in Figure 1 and includes the careful selection and identification of important search words as well as search terms to be used to find the most suitable journal articles.

Google advanced search is useful for identifying gray literature and is widely used; however, search results can be incomplete, and the process is time-consuming. It has been demonstrated that manually searching for relevant target documents on the websites of specific organizations is more efficient than using Google advanced search. To ensure the comprehensiveness of the search strategy, both search methods were used for documents published between June 1, 2017, and May 31, 2022, both dates inclusive. This period was chosen to reflect the current state of evidence and thought regarding health system sustainability. During a search of peer-reviewed literature in PubMed, key search terms were identified.

In addition to the targeted manual searches, Google advanced search was utilized to conduct searches using the search terms listed in Table 1. The reference sections of publications pertaining to the sustainability of healthcare systems were combed for additional documents using a snowballing strategy.

**Table 1:** Google advanced search and key strategies.

Exact word or phrase		Any of these words
Healthcare industry in the UK	AND	Sustainable or Resilient
Health practices	AND	Current
Surgical industry in the UK	AND	Determining factors
Healthcare practices	AND	Determining factors
Surgical industry of the United Kingdom	AND	Sustainability

The PRISMA guidelines were used as an overarching framework for screening and selecting documents. A PRISMA flow chart was created that detailed document identification, screening, and inclusion. Finally, to achieve the research goals and objectives, 15 journal articles were chosen for thematic analysis and discussion.

Study Criteria

The selected documents were limited to book chapters, reports, policy statements, government policy documents, and submissions published online or in print in English or could be translated into English. Opinion pieces published in peer-reviewed journals were also considered. The publication window was extended from May 2017 until June 2022.

Relevant articles were those that specifically addressed the sustainability and performance of the healthcare system, including threats, challenges, and sustainability drivers; frameworks or policy responses for improved sustainability; and the formulation, implementation, and evaluation of interventions for improving sustainability. Articles whose primary focus was on the diagnosis or management of disease outcomes were excluded, as were population health prevention initiatives.

Data Extraction

Due to the nature of the study, the theme synthesizing technique was used to analyze the findings of the 15 selected journal articles using Mendeley Reference Manager. The arrangement of themes is depicted in Table 2 based on the preliminary assessment.

**Table 2:** Theme structure based on the preliminary assessment.

Variables	Themes
1. The current sustainable healthcare practices in the surgical industry in the United Kingdom	Waste management practices
	Carbon footprint
	Water conservation
	Air pollution management
	Supply chain
2. Key determining factors of high-quality treatment in the surgical industry in the United Kingdom	Healthcare resource management. Ethical purchase. Business service and integration. Hospital stays. Mortality and morbidity
3. Critically evaluate how to incorporate novel sustainable healthcare practices into the surgical industry to provide the highest quality surgical care in the United Kingdom	Reducing solid waste
	Environmentally friendly purchasing
	Water conservation
	Care pathway and travel
	Leadership and cultural change

Data Synthesis

A single author performed data synthesis. Given the large number of articles and data likely to be included, the potentially low quality of the studies, and the expected amount of missing data, conclusions were interpreted with caution. Based on the sample size and data distribution, each study was awarded a weight. These rates were eventually combined and displayed in the same manner and qualitative analysis was done.

Results

**Figure 2:**
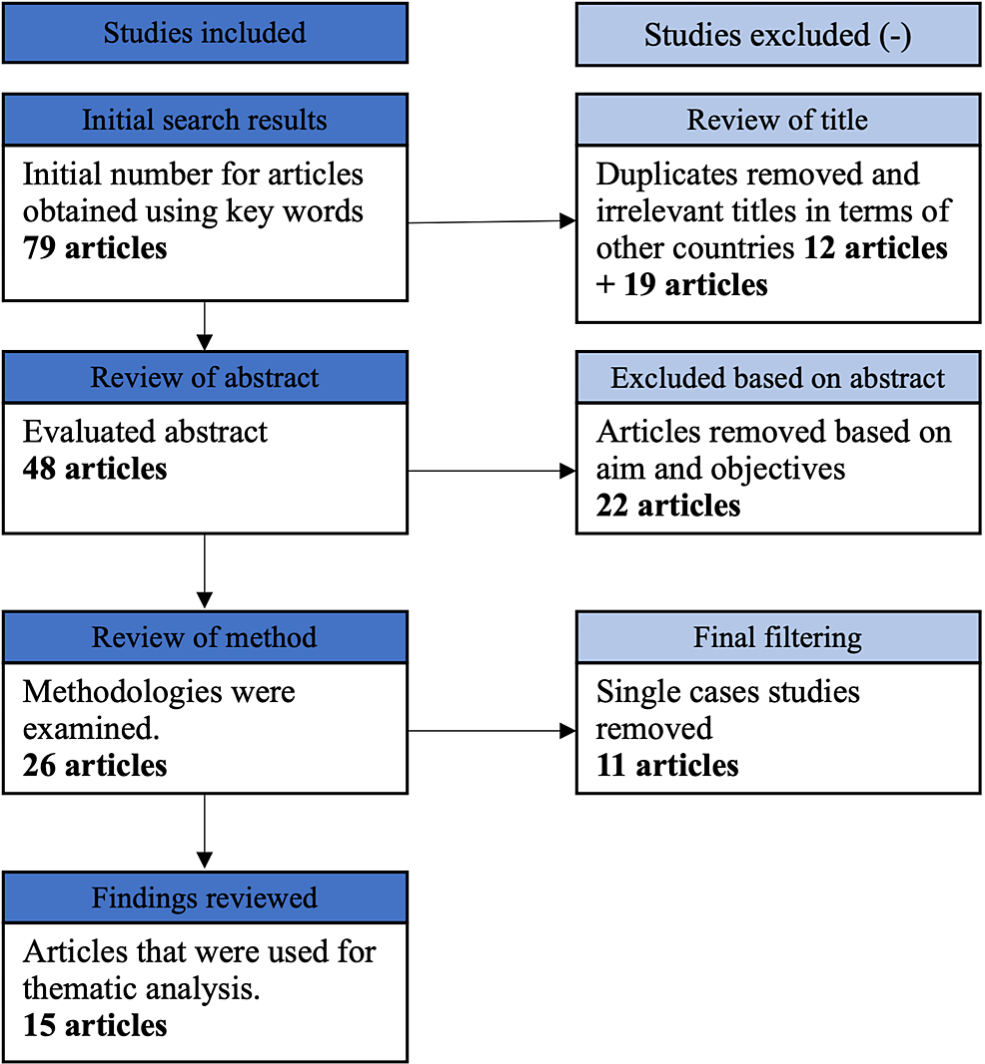
PRISMA flow chart. PRISMA: Preferred Reporting in Systematic Reviews and Meta-Analyses

The predefined literature search yielded 79 results. Following the removal of 12 duplicates, 19 studies were excluded based on their title, abstract, and keywords. Following a thorough review of the full texts, 64 studies were excluded for the reasons listed in Figure 2. Finally, 15 articles were selected for analysis. The themes were then applied to the selected articles.

According to the thematic analysis shown in Table 3, 10 articles evaluated some existing sustainability practices. Only seven articles discussed significant determinants of quality healthcare. Only 86.67% (n = 12) of articles highlighted the implications of sustainability. The key predictors of high-quality medical care are effective resource management, the acquisition of a moral surgical team, the provision of professional services, integration, short hospital stays, and low mortality and morbidity rates. Conserving water, optimizing treatment routes and transportation, and creating cultural change were found to be the pillars of high-quality, sustainable healthcare.

**Table 3:** Thematic analysis.

		Research 1	Research 2	Research 3	Research 4	Research 5	Research 6	Research 7	Research 8	Research 9	Research 10	Research 11	Research 12	Research 13	Research 14	Research 15	
Variables	Themes	Design opportunities to reduce waste in operating rooms	Design opportunities to reduce waste in operating rooms	Operating room greening initiatives—the old, the new, and the way forward	Sustainable surgery: in and out of the operating theater	The carbon footprint of surgical operations: a systematic review	Minimizing carbon and financial costs of steam sterilization and packaging of reusable surgical instruments	The impact of surgery on global climate: a carbon footprinting study of operating theaters in three health systems	Operating in a climate crisis: a state-of-the-science review of life cycle assessment within surgical and anesthetic care	Effectiveness of architectural separation of septic and aseptic operating theaters for improving process quality and patient outcomes: a systematic review	Sustainability in healthcare: efficiency, effectiveness, economics, and the environment	“Every breath we take: the lifelong impact of air pollution"—a call for action	Navigating the sustainability landscape: a systematic review of sustainability approaches in healthcare	Built to last? The sustainability of health system improvements, interventions, and change strategies: a study protocol for a systematic review	The green print: advancement of environmental sustainability in healthcare	Evidence-based intervention sustainability strategies: a systematic review	Analysis
	Waste management practices	Between 50–70% of the hospital’s trash is generated in operating rooms and labor and delivery rooms	Previous studies have found that the waste produced in operating rooms is substantially greater than the waste produced in other departments, accounting for up to 33% of the garbage produced overall	In affluent nations, hospitals produce, on average, 1% of the nation’s solid waste	-	-	-	-	Across a variety of environmental factors, single-use surgical equipment was generally more damaging than comparable reusable equipment	-	-	-	-	-	Modern healthcare is a significant source of air pollutants that are harmful to human health	-	5
	Carbon footprint	Around 9.8% of the nation’s emissions were caused by the healthcare business		In the United Kingdom and the United States, greenhouse gas emissions have reached 3% and 10%, respectively. Operating rooms contribute dramatically to these emissions due to the use of energy-intensive medical equipment, the discharge of anesthetic chemicals into the atmosphere, specialized illumination, and a 24-hour operating schedule	An estimated 4–5% of England’s carbon footprint can be attributed to the country’s healthcare system. Although only a small portion of surgical practice, the operating room’s annual carbon footprint in one UK teaching hospital was comparable to the energy use of more than 2,000 households	The carbon footprint of one operation varied from 6 to 814 kg CO_2_ equivalents	The carbon footprint of cleaning and packaging instruments was the lowest when they were sold in sets and increased by two to three times when sold separately	The energy demands for heating, ventilation, and air conditioning in operating rooms were discovered to be three to six times higher than those of the hospital as a whole. An estimated 9 million tons of CO_2_e are emitted annually as a result of surgery in the three nations under study	Operating surgical suites had an annual CO_2_e effect of between 3,200,000 and 5,200,000 kg	-	-	-	-	-	-	-	7
1. The current sustainable healthcare practices in the surgical industry in the United Kingdom	Water conservation	-	-	-	-	-	-	-	-	-	-	-	-	-	According to estimates, 150,000 people die each year owing to health risks brought on by climate change, such as social unrest, food and water insecurity, food- and water-borne infections, decreasing air quality, and extreme weather events	-	1
	Air pollution management	-	-	-	-	-	-	The main contributors to greenhouse gas emissions were anesthetic gases and energy use	The primary emission hotspots found in the operating room and procedure-specific evaluations were anesthetic gases, single-use equipment, and heating, ventilation, and air conditioning system operation	-	-	Because of the steadily rising usage of fossil fuel-powered cars, air pollution has emerged as one of the biggest threats to human health. The Clean Air Acts of the 1950s and 1960s were supposed to have reduced the health dangers associated with air pollution, but the situation with air pollution in the United Kingdom has since become worse to the point where it is now responsible for 40,000 more fatalities per year	-	-	-	-	3
	Supply chain	-	The study discovered two waste hotspots: improper sorting of hazardous and non-hazardous medical waste and the logistical packing (tertiary, secondary, and primary) of goods	-	-	The purchase of consumables and power were the two biggest carbon hotspots in the operating rooms under investigation	Streamlining sets resulted in higher carbon and financial expenses	-	-	-	-	-	-		-	-	3
	Healthcare resource management	-	-	The WHO estimates that 85% of hospital waste is non-hazardous, 10% is infectious, and the remaining 5% is non-infectious yet harmful. Unfortunately, up to 90% of this common trash is mistakenly classified as “clinical” or hazardous waste in the operating room, which has negative effects on recycling and increases expenses to properly treat and dispose of the waste by up to 20 times	The surgical community’s response to the COVID-19 pandemic has shown that it is capable of quickly changing surgical practice in response to an international emergency. Surgeons are well-positioned to push for healthcare that is low-carbon and climate resilient	-	-	-		-	-	-	-	-	-	-	2
	Ethical purchase	-	-	-	-	-	-	-	-	-	-	-	-		-	-	0
2. Key determining factors of high-quality treatment in the surgical industry in the United Kingdom	Business service and integration	-	-	-	-	-	-	-	-	-	-	-	-	Demands for greater standards of patient safety and quality of care as well as lower costs have been sparked by the introduction of new medical technology, including new diagnostic tests, medications, medical equipment, and digital healthcare services	-	-	1
	Hospital stay	-	-	-	Implement policies that promote early ambulatory diagnosis, early release, and short hospital stays. A service supplied by a consultant is necessary	-	-	-	-	-	-	-	-		-	-	1
	Mortality and morbidity	-	-	In many different ways, climate change will have a negative impact on health. For air pollution and heart illness, airway disease, as well as elevated vector-borne and rodent-borne infections, there is a clear, linear dose-response curve	-	-	-	-	-	-	Sustainable healthcare delivery ensures that the proper patients are operated on as well as that those who are do so for a shorter amount of time, lowering hospital expenses and subsequent postoperative morbidity	-	-	The sustainability of the healthcare system is also challenged by aging populations and the constantly rising burden of chronic illnesses	Nine million premature deaths worldwide in 2015, or 16% of all deaths, were caused by pollution, which is a primary source of morbidity and mortality	-	4
	Reducing solid waste	-	Reuse of secondary and tertiary packing, reuse of textiles and protective apparel, reuse of sterilizing packaging, reuse of equipment, and the dissemination of cleanliness information to enable future use; clarifying the distinction between NHMW and HMW, using product tagging to facilitate effective sorting, collecting PP and paper from different packaging separately, and optimizing the contents of surgical sets are all examples of waste sorting optimization	Several types of plastic, glass, and paper are simple recyclables. Other products, including batteries, linen, steel, surgical towels, gowns, back table coverings, stainless steel basins, and Mayo stand covers, may also be recycled where it is practical	It is important to critically evaluate emerging technology from a green perspective, such as minimally invasive surgery and robotic surgery	Future studies should concentrate on minimizing the operating room’s carbon footprint by streamlining procedures, extending analyses to other surgical settings, and identifying carbon hotspots as potential targets for reduction	-	-	-	-	-	-	-	-	-	-	4
	Environmentally friendly purchasing	-	-	Prefilled syringes can cut down on medication and plastic waste. Surgical packs can also be changed, and reusable alternatives can be used in place of disposable towels, handles, and basins. If lined with disposable liners, plastic suction bottles can be reused. Reusable sharps containers can take the place of disposable ones. Hard metal cases can be used in place of the blue sterile wrap. You can use reusable alternatives in place of disposable surgical gowns and linen drapes	-	By employing reused or reprocessed surgical instruments and streamlining procedures, it is possible to lower the carbon footprint of surgery	-	-	-	-	-	-	-	-	-	-	2
3. Critically evaluate how to incorporate novel sustainable healthcare practices into the surgical industry to provide the best quality treatment in the surgical industry in the United Kingdom	Water conservation	-	-	-	-	-	-	-	-	-	-	-	-	-	-	-	0
	Care pathway and travel	-	-	-	Travel for patients must be kept to a minimum. More people need to use outreach clinics that offer telemedicine, video conferencing, day procedures, minor surgery, and endoscopy	-	-	-	-	-	The size of the NHS workforce, as well as how we commute, provide care, and obtain materials, all have a substantial environmental impact	Require safer alternatives to the “school run,” increased safe cycling networks, bicycle education in schools, employer support programs for car-free commuting, promotion of leisure cycling, and urban corridors and islands for safer walking and bicycling	-		-	-	3
	Leadership and cultural change	Surgeons are particularly positioned to lead initiatives to enhance the environmental sustainability of the operating rooms due to their resource-intensive job in the operating rooms	-	Leadership in this context entails the use of already-existing rules and regulations that support operating room greening, regular application and auditing of greening programs, as well as a culture that is supportive of reducing waste. The policies must first be in place to provide direction for staff since healthcare workers are required to abide by the policies and guidelines of the hospital where they are employed regarding waste management	Whether or not surgery is necessary must be critically evaluated by surgeons. In cases of advanced cancer, salvage surgery may be used as a form of palliative therapy	-	The preparation of instruments as sets, the incorporation of separately wrapped instruments into sets as opposed to simplifying them, effective machine loading, and the use of low-carbon energy sources in addition to recycling can all result in carbon and monetary savings	Without sacrificing patient safety, emissions reduction techniques such as avoiding desflurane and utilizing occupancy-based ventilation may be able to decrease the impact of surgical services on the environment	-	Evidence supporting or opposing the architectural separation of a septic or aseptic operating room was not found during the review. Particularly, there is no proof that architectural separation has a negative effect	While providing care over the phone or through telemedicine is obviously not appropriate for everyone, habitually using new or even basic digital technology may have wider societal and environmental benefits that, if not implemented, may result in avoidable risks for all	Many of the most important health problems of our time, including asthma, chronic obstructive pulmonary disease, cancer, heart disease, stroke, diabetes, obesity, and even dementia, are influenced by air pollution. It is obviously our responsibility to speak up and be adequately informed on the subject to promote awareness among the public when the public and patients are subjected to such a clear and preventable cause of death, disease, and disability	Similar structures for sustainability were discovered, demonstrating that general learning may be obtained from across settings to inform sustainability processes and research, despite the fact that many approaches were established inside particular interventions and settings	Through creative projects, development programs, and interventions—hereafter referred to as “programs”—change is implemented to improve the sustainability of the healthcare system more frequently closer to the front lines of service	To more accurately assess clinical materials and processes and to support the creation of performance indicators to direct and monitor progress, a complete approach to healthcare environmental emissions research is required, including the development of analytical methodologies and tools	Literature that emphasizes maintaining public health EBIs ought to provide the notion with a clear definition. Improved documentation of the methodology used, the steps taken, and the modifications needed to keep the intervention going could help standardize and advance the idea	12

The Royal College of Surgeons, the premier surgical organization in the United Kingdom, promotes sustainability in operating rooms through five key initiatives, namely, cutting back on wasteful purchases, conserving water, improving care pathways and transport, and fostering cultural change [4].

By streamlining operations, expanding the analysis to different surgical settings, and identifying carbon hotspots as potential targets for reduction, the future of medicine should focus on reducing the operating room’s carbon footprint [3]. To reduce solid waste, it is vital to assess new technology from a green standpoint, such as minimally invasive surgery and robotic surgery [5]. Simple recyclables include various plastic, glass, and paper varieties. Where possible, other materials can also be recycled, such as batteries, linen, steel, surgical towels, gowns, back table covers, stainless steel sinks, and Mayo stand covers [6].

The carbon footprint of surgery can be reduced by using recycled or reprocessed surgical instruments and streamlining operations [3]. Syringes that are already loaded with medication and plastic might reduce waste. Reusable alternatives can be utilized in place of disposable towels, handles, and basins, and surgical packs can also be changed. Plastic suction bottles can be reused if they are lined with disposable liners. Reusable sharps containers can be utilized instead of disposable ones. The blue sterile wrap can be replaced by hard metal cases. You can use reusable alternatives to disposable surgical gowns and linen drapes [6].

Every day, large volumes of water are used in surgery; however, this resource is utilized inefficiently and carelessly discarded. Using alcohol-based products might save millions of liters of water annually. We can help the environment by lowering the amount of energy needed to treat and transport water, challenging the use of excess water, and reducing water consumption. It can also help hospitals reduce operational costs by reducing the need to wash and prepare towels, among other things [4].

There is a significant environmental impact associated with the size of the National Health Service workforce, as well as their commuting habits, care delivery methods, and supply procurement [7]. Patients should only travel if it is absolutely necessary. It is important to increase the use of outreach clinics that offer telemedicine, video conferencing, day treatments, minor surgery, and endoscopy [5]. Increased safe cycle networks, bicycle instruction in schools, employer support programs for car-free commuting, the promotion of leisure cycling, and urban corridors and islands for safer walking and bicycling are all necessary safer alternatives to the commute [8].

Due to the resource-intensive nature of their work in operating rooms, surgeons are uniquely qualified to lead efforts to improve the environmental sustainability of operating rooms [9]. In this context, leadership is exemplified by the use of existing norms and regulations that enable operating room greening, regular implementation and auditing of greening programs, and a culture that supports waste reduction. Because healthcare workers are obligated to follow the hospital’s waste management rules and regulations, waste management policies must be in place to guide staff [6]. Reducing needless procedures will reduce the effects that follow, but surgeons must thoroughly assess whether surgery is necessary. Salvage surgery may be performed as a palliative therapy in cases of advanced cancer [5]. Carbon savings and financial savings can be achieved by preparing instruments as sets, incorporating separately wrapped instruments into sets as opposed to their simplification, ensuring effective machine loading, using low-carbon energy sources, and recycling. Even though many strategies were developed inside specific interventions and contexts, similar structures for sustainability were found, suggesting that general learning may be gleaned from various settings to guide sustainability processes and research. Thus, by using occupancy-based ventilation and eliminating desflurane, for example, surgical services may be able to reduce their environmental impact without compromising patient safety [10]. Using new or even simple digital technology regularly may have wider societal and environmental benefits that, if not implemented, may result in an avoidable risk to all. As not everyone can benefit from telemedicine or care delivered over the phone, this practice has obvious limitations [7]. Furthermore, air pollution impacts many of the most significant health issues of our time, such as asthma, chronic obstructive pulmonary disease, cancer, heart disease, stroke, diabetes, obesity, and even dementia. When the public and patients are exposed to such a clear and preventable cause of death, disease, and disability, it is the responsibility of the leaders of the healthcare system to speak up and be sufficiently informed on the subject to promote awareness among the general public [8].

The significance of sustainability was recognized in every study, but the concept of sustainability varied between these studies. However, none of the 15 research articles examined the topics of water conservation or ethical shopping. Only one study discussed business services, mortality, and morbidity. There is significant duplication of data among the researched studies on mortality, morbidity, and business services [1,3,5-17].

Discussion

The UK healthcare system’s carbon footprint is around 4-5%, and one of the trending sustainable healthcare practices is the use of reusable instruments in theaters to reduce waste from single-use products. The theater industry is a significant contributor to waste in the healthcare system. The surgical community has adopted alcohol-based hand rubs to reduce water waste from hand washing. Anesthetic drugs are the main impediment to sustainability in the medical sector, as they emit a majority of greenhouse emissions during surgical procedures. The surgical industry maintains appropriate shipping and packaging practices to exhibit sustainability in its supply chain [2].

Effective resource management, moral surgical team purchases, business services and integration, short hospital stays, and low mortality and morbidity rates are determinants of high-quality medical care. Adapting surgical procedures in response to the pandemic has demonstrated resilience in the healthcare sector [5]. Ethical healthcare product creation, procurement, and purchasing should consider high-quality medical services and a smaller carbon footprint [2]. Awareness of labor rights abuses in the surgical supply chain is necessary for the health sector. The introduction of new medical technology has sparked demands for greater standards of patient safety, quality of care, and lower costs.

To promote sustainability in high-quality healthcare, unnecessary spending should be reduced, water conservation should be prioritized, care pathways and transportation should be enhanced, and cultural change should be promoted. The operating room’s carbon footprint can be reduced by streamlining procedures, extending the analysis to various surgical settings, and identifying carbon hotspots as possible reduction targets. Surgery can lower its carbon impact by using recycled or reprocessed surgical tools and optimizing procedures [5]. Misuse of water in surgery contributes to increased water-borne illnesses and water waste, and decreased water usage can improve the environment by lowering the energy required to process and transport water. Patients should only travel when necessary, and outreach clinics should offer telemedicine, video conferencing, day treatments, minor surgery, and endoscopy [7]. Surgeons should take on sustainable leadership roles to transform the culture and strike a balance between the managerial and clinical components of surgery.

Limitations and recommendations

While the reported findings have important implications for public health research, the author considers the systematic review’s limitations. The review only included studies on sustainability that were published in peer-reviewed journals. Gray literature and unpublished literature were excluded due to the possibility of publication bias. The significance of sustainability was recognized in every study, but the concept of sustainability varied across these studies. However, none of the 15 articles examined the topics of water conservation and ethical shopping. Business services, mortality, and morbidity were discussed in only one study. The limitation of data among the researched studies on mortality and morbidity and business services poses the largest limitation to this study.

Another limitation of this review is that the majority of the screening, data extraction, and coding were performed by one author. Although duplicate data extraction is generally advised in systematic reviews, it is acknowledged that due to time and resource limitations, this is not always practicable. This could have led to incomplete or inaccurate information being gathered or biased inclusion or exclusion. The fact that the author did not use an existing technique for evaluating the quality of the work makes it impossible to judge the worth or correctness of the constructions from each approach. While the author was able to guarantee that each paper had at least the necessary amount of data to effectively describe the technique owing to the quality standards outlined in the data extraction form, it did not evaluate the quality of the approaches themselves.

To determine their relevance and the need for additional development, many of the approaches discussed in this review suggest that they be implemented and assessed further in various healthcare projects and settings. Future research in this area should concentrate on putting the current strategies to use to comprehend the application procedures and evaluate the overall effects of their implementation. The continuation of evidence-based medicine in contexts other than that of public health initiatives should be reported in future studies.

## Conclusions

The concept of sustainability refers to the ability of a system to persist over time while maintaining its essential characteristics and functions. The use of sustainability to improve the quality of the surgical industry was analyzed.

The novel practices to improve the quality of sustainability included changes in reducing solid waste, usage of environmentally friendly purchasing, new methods of water conservation, care pathway and travel, and, finally, leadership and cultural change. Reduced mortality, morbidity, and business services have a major impact on the surgical industry’s sustainability. The largest damaging influence on sustainability in this industry is attributed to anesthetic gas emissions from operating rooms. However, there was a large gap between the existing statistics and their implications, indicating that more research is required to properly understand the impact of anesthetic gas emissions and other factors on the surgical industry’s sustainability.
